# Challenges for implementation of inter-sectoral efforts to improve outbreak response using consolidated framework for implementation research; Iran’s COVID-19 experience

**DOI:** 10.1186/s12913-022-08510-4

**Published:** 2022-09-03

**Authors:** Marjan Mirzania, Elham Shakibazadeh, Mahnaz Ashoorkhani

**Affiliations:** Department of Health Education and Promotion, School of Public Health, Tehran University of Medical Sciences, Tehran, Iran

**Keywords:** Inter-sectoral collaboration, Responsiveness, Health system, COVID-19, CFIR, Qualitative study, Iran

## Abstract

**Background:**

The recurrence of emerging infectious diseases reminds us that rapid response to related outbreaks require coordinated inter-sectoral/ organizational and trans-disciplinary approaches. This study examined the challenges for implementation of inter-sectoral efforts to improve COVID-19 pandemic response in Iran using the consolidated framework for implementation research (CFIR).

**Methods:**

We conducted a qualitative content analysis of in-depth interviews between March 2020 and February 2021 in Tehran, Iran. Participants included health professionals and experts involved in the prevention, treatment and control of COVID-19 pandemic from different levels of the health system (macro: Ministry of Health and Iranian National Institute of Health Research; meso: universities of medical sciences and health services; and micro: hospitals), selected using purposive sampling. Recorded interviews were transcribed verbatim and coded using a deductive approach (CFIR constructs).

**Results:**

In total, 12 interviews with the participants were conducted; and eight themes emerged as the most important challenges for implementation of inter-sectoral efforts to improve outbreak response in COVID-19. These challenges include lack of proper intervention sources, complexity, poor networking with external organizations, cultural issues, inadequate availability of resources, inadequate access to knowledge and information about inter-sectoral collaboration implementation, and planning issues for it.

**Conclusions:**

Implementing inter-sectoral efforts to improve outbreak response require addressing several implementation challenges. There should be effective leadership and command system, prioritizing the problem, having proper intra-sector collaboration, adequate supervisory, strong social capital, managers and officials’ positive beliefs and organizational culture towards inter-sectoral collaboration, sufficient knowledge and information about the implementation, and providing proper programs to implement inter-sectoral collaboration. These findings recall the need to develop and in particular, implement a specific infra-structure through a well-designed program at the government level to strengthen this approach.

**Supplementary Information:**

The online version contains supplementary material available at 10.1186/s12913-022-08510-4.

## Background

Over the past few decades, the world has witnessed transnational spread of emerging infectious diseases (EIDs) due to population growth, urbanization, and globalization [1]. The coronavirus disease 2019 (COVID-19) outbreak is currently the largest global crisis, first reported in December 2019 in Wuhan, China [1, 2]. As of August 2022, more than 600 million confirmed cases; including more than 6 million deaths were reported worldwide [3]. In Iran, according to the Ministry of Health and Medical Education (MOHME), the first cases of COVID-19 were identified on 19 February 2020 [4]. And as of this writing (27 August 2022), over 7 million confirmed cases including more than 144,000 deaths have been reported [3].

The recurrence of EIDs such as COVID-19, severe acute respiratory syndrome (SARS), Ebola, and Avian Influenza reminds us that rapid response to these outbreaks requires coordinated inter-sectoral/ organizational and trans-disciplinary approaches. As the world gets closer, EIDs are becoming a greater threat; and necessitates the need for coordination and collaboration at local, regional and international levels [5]. The need for inter-sectoral collaboration (ISC) is not a new issue. It was highlighted in both the “Health for All 2000” in the Alma-Ata Declaration in 1978, and the “health promotion strategies” in Ottawa Charter in 1986 [6]. The term ISC refers to collective actions in which more than one agency plays different roles for a common purpose [7]. In 1997, World Health Organization (WHO) introduced the concept of “inter-sectoral action for health”- “A recognized relationship between part or parts of the health sector with part or parts of another sector which has been formed to take action on an issue to achieve health outcomes”- as the cornerstone of health for all in the twenty-first century [8]. The 2005 Bangkok Charter re-emphasized the health promotion through the ISC, and finally the European Union (EU) in 2006 emphasized the “Health in All Policies” approach [9]. All this international evidence shows that ensuring community health requires ISC and participation [10]. In the COVID-19 crisis, the need for collaboration across all sectors of society in order to manage its health, social and economic impacts was also revealed [5, 11]. WHO recommended all sectors to unit their strengths to overcome the crisis [12]. Although, the necessity of ISC in management of COVID-19 has been highlighted in literature [11, 13], efforts to implement ISC have often been described as challenging [14, 15]. Some existing challenges are lack of commitment and/or poor leadership, tensions and mistrust between actors, holding the own power and refusing to compromise, strict regulations, lack of knowledge on inter-sectoral issues, inadequate coordination and communications, and limited financial and human resources [15–19].

In recent years, the implementation science (IS) has been considered as an important discipline to study the research-to-practice gap and accelerate the implementation of evidence-based interventions in the “real world” [20]. IS has been defined as “the scientific study of methods to promote the systematic uptake of research findings and other evidence-based practices into routine practice, and, hence, to improve the quality and effectiveness of health services” [21]. Various theoretical frameworks have been defined for implementation science research, one of which is the consolidated framework for implementation research (CFIR) [22]. CFIR includes five main domains including intervention characteristics (key characteristics of an intervention), outer setting (economic, political, and social context of the intervention), inner setting (structural, political, and cultural contexts), characteristics of individuals (cultural and organizational norms of individuals involved in the intervention), and process of implementation (the strategies that might influence implementation) with 39 constructs and sub-constructs [22].

In this study, we opted to use the CFIR because, first, it is a comprehensive combination of implementation theories and models that designed as a roadmap to guide the systematic evaluation of multi-level implementation of interventions and to identify factors that may affect the implementation of the intervention and its effectiveness [22]. Furthermore, it is a flexible framework that can be easily customized in different settings and contexts [23]. Despite the importance of ISC in management of COVID-19 pandemic, it seems that no attempt has been made to identify the challenges of its implementation using a comprehensive framework. Therefore, this study was conducted with the aim of exploring the challenges for implementation of inter-sectoral efforts to improve COVID-19 pandemic response in Iran using CFIR. Identifying the challenges associated with the successful implementation of ISC efforts to improve accountability in the face of biological crises can be a roadmap for planning to improve responsiveness in the future.

## Methods

### Study design and setting

We conducted a qualitative content analysis of semi-structured and in-depth interviews between March 2020 and February 2021 in Tehran, Iran. This study followed the consolidated criteria for reporting qualitative research (COREQ) [24] (see Additional file 1).

Participants included experts from different levels of the health system involved in the prevention, treatment and control of COVID-19 pandemic. To access the list of experts, we first listed and reviewed the related health organizations and then selected the participants purposively and using the brainstorming method. The eligible participants were selected from three macro (MOHME: three individuals; and Iranian National Institute of Health Research (INIHR): one individual), meso (universities of medical sciences and health services: six individuals) and micro (hospitals: two individuals) levels; in order to have a comprehensive understanding from the different level lenses. In macro level, participants were recruited from the Infectious Diseases, and the Health Education and Promotion Offices of the Ministry of Health; and the INIHR. The MOHME offices were responsible for managing, supervising, facilitating, and regulatory actions in the fields of prevention, treatment, and public education nationwide. The INIHR is also a nationwide institution responsible for watching out the pandemic status, as well as production and promotion of the scientific evidence required for health planners and policy-makers in order to make scientific cooperation between similar domestic and foreign organizations, networking, and empowering researchers and policy-makers to meet the needs of health system to control the pandemic. In meso level, we recruited researchers from the universities that were responsible for providing evidence, providing the related guidelines and protocols, providing public education content that were mostly broadcasted through media. We also interviewed with mid-level managers working at health and treatment deputies of the universities that supervised field work activities. In micro level, we recruited head of the hospitals and treatment managers of the hospitals that were mostly responsible for treating patients with COVID-19. In order to meet the ethical principles, invitations were sent to the individuals to participate in the study. Inclusion criteria were having at least five years of work experience, being involved in COVID-19 management, and willingness to participate in the study. Attempts were made to have the maximum variation among participants according to age, education level, work experience and organizational role and responsibility. Three experts refused to participate in the study due to time limitations. Due to the COVID-19 pandemic situation, five participants preferred to have online interviews (via WhatsApp or Skype video call); while others (*n* = 7) participated in face-to-face interviews set in their workplaces (where only the participants and the researchers were present).

### Interviews

The initial interview guide was provided based on sample interview questions available at http://CFIRguide.org [25]. Damschroder et al. (2009) suggested that researchers could select the constructs of CFIR that are most relevant to their study setting [22]. Accordingly, we reviewed the interview guide and selected study-related constructs (see Additional file 2). Then, we pilot tested it in one test interview. The interviews were conducted by two female researchers specialized in Health Education and Promotion (MM; PhD candidate, and ESh; Professor), who have good experience of conducting qualitative studies. They had no prior relationship with the participants. At the beginning of each interview, participants were explained about the objectives of the study, the assurance of confidentiality, the voluntary participation, and the freedom to withdraw at any time. Also, written consent and permission for audio recording were obtained from all participants. Each interview lasted between 30 to 50 minutes. At the end of each interview, demographic information of the participants was collected (i.e. age, gender, education, specialty, work experience, and organizational role). The interviews continued until data saturation was reached. After 10 interviews, the data were repeated, but for more assurance, data collection was continued until the 12th interview. No repeat interviews were performed. Field notes were taken after the interviews.

### Data analysis

The interviews were recorded and transcribed verbatim on Microsoft Word by MM and MA. The transcripts were uploaded to the MAXQDA 18 software for analysis. MM and ESh read the transcripts and independently coded the data using a deductive approach (CFIR constructs) (investigator triangulation). Then, coding was compared and discrepancies discussed. Trustworthiness of the data was assessed using Lincoln and Guba criteria [26]. The credibility of the study was ensured by allocating adequate time to data collection and analysis, and recruiting participants with maximum diversity. Confirmability of the data was ensured by providing coded interviews to the participants for comment and/or correction. Also, to ensure dependability, two authors analyzed the data independently. Furthermore, transferability was obtained by reviewing and confirming the process of data analysis by a researcher outside the study but familiar with qualitative research methods.

## Results

A total of 12 participants were interviewed. Ten (83.3%) participants were male. The mean age was 50.66 years (SD = 6.95) (range 40 to 65 years). Four participants (33.2%) were working in the MOHME and INIHR, six (49.8%) were working in the medical universities, and two (16.6%) were working in the hospitals. Most of them (75%) had more than 15 years of experience. Participants’ characteristics are presented in Table 1.

**Table 1:** Characteristics of the participants (*n* = 12)

Characteristic	n	%
Gender		
Male	10	83.3
Female	2	16.7
Age (years)		
< 35–45	3	25
46–55	6	50
56+	3	25
Mean ± SD = 50.66 ± 6.95		
Education		
Ph.D	8	66.7
M.D	4	33.3
Specialty		
Health Policy	1	8.3
Epidemiology	3	25
Health Education and Promotion	2	16.7
Medical Doctor	5	41.7
Health in Emergencies and Disasters	1	8.3
Organization		
Infectious Diseases Office/ MOHME^a^	2	16.6
Health Education and Promotion Office/ MOHME	1	8.3
INIHR^b^	1	8.3
Faculties/ Medical universities	4	33.2
Health Deputy/ Medical universities	2	16.6
Hospital	2	16.6
Work experience (years)		
< 15	3	25
15+	9	75

^a^*MOHME* Ministry of Health and Medical Education; ^b^*INIHR* Iranian National Institute of Health Research

### Identified challenges based on CFIR domains and constructs

The participants commented around eight constructs of the CFIR in five domains (intervention characteristics, outer setting, inner setting, characteristics of individuals, and process of implementation) related to the implementation of ISC efforts to improve the response to COVID-19 pandemic (Table 2).

**Table 2:** Challenges of implementation of inter-sectoral efforts to improve COVID-19 pandemic response; Categories, themes and sub-themes

Domains and Themes	Sub-themes
**Intervention characteristics**	
Intervention source	Establishment of COVID-19 headquarter
	Developing protocols and guidelines
	Producing scientific evidence
Complexity	Leadership in managing COVID-19 pandemic
	United command
	Prioritizing COVID-19 pandemic
	Intra-sectoral collaboration
	Supervising related organizations activities
	Gap between prevention and treatment
	Social capital
**Outer settings**	
Cosmopolitanism	Networking with external organizations
**Inner setting**	
Culture	Manager and officials’ beliefs
	Non-institutionalization of the culture of inter-sectoral collaboration
Available resources	Available resources to implement inter-sectoral efforts
Access to knowledge and information	Access to knowledge and information towards implementation of inter-sectoral efforts
**Characteristics of individuals**	
Knowledge and beliefs about the intervention	Awareness and beliefs towards inter-sectoral collaboration
**Process of implementation**	
Planning	Structures and programs for inter-sectoral collaboration

### Domain 1: intervention characteristics

Participants highlighted intervention source and complexity as key challenges for the successful implementation of ISC efforts to improve the response to COVID-19 pandemic.

#### Intervention source

Intervention source stands for the perception of key stakeholders about whether the intervention (here, inter-sectoral efforts) is externally or internally developed [22]. The *formation of the National Corona Headquarter* and its provincial branches made the different organizations to collaborate in order to respond to the pandemic; however there were implementation challenges in this regard: *“There is not good collaboration in implementation of the decisions and plans announced by the committees of COVID-19. I think this is an important obstacle for Iran’s response to COVID-19.” (Participant at Hospital with 12 years of work experience; Male; 42 years old).*

Participants reported that their organizations were automatically involved in COVID-19 pandemic management through informing and educating the public, developing protocols and guidelines, producing scientific evidence, publishing articles related to the specialized fields, and providing medical services to the patients. It seems that assigning responsibilities from upstream organizations to make ISC was rarely clear to most participants working in the field. At all level organizations, different committees had been formed within various sections to take part on the task of producing scientific evidence and public education content. Our data showed that the need to inform and educate the public was spontaneously identified in various organizations and each organization undertook this task independently. Several mobilizations and campaigns were created in different organizations that worked independently. Therefore, macro level decisions and assigning roles and dividing tasks to different organizations/individuals; and/or ISC (between different organizations or universities) were rarely observed. ISC to *develop protocols and guidelines* was more collaborative; and participants from MOHME stated that they coordinated the efforts with universities to prepare related guidelines and protocols: *“We coordinated with faculty members to prepare related guidelines.” (Participant at MOHME with 10 years of work experience; Female; 40 years old).*

Moreover, some organizations, especially universities, started to produce scientific evidence for decision-makers and health program planners: *“Some faculty members were involved in various committees to produce scientific evidence or give advice to the policymakers.” (Participant at University with 18 years of work experience; Male; 52 years old).*

Participants stated that their efforts to respond to inter-sectoral efforts to control the pandemic were from both internal and external sources, however, internal source (spontaneous and voluntary action) has played more prominent role than external source (determination and division of tasks from upstream): *“There were both; it means that much of what we started and carried out in the first months of the pandemic was voluntarily, such as informing and educating the public or producing protocols and guidelines, but over time many orders came from the Ministry of Health.” (Participant at INIHR with 15 years of work experience; Male; 50 years old).*

#### Complexity

Complexity refers to the perceived difficulty of the intervention (ISC), reflected by duration, scope, radicalness, disruptiveness, centrality, and intricacy and number of steps required to implement [22]. The participants stated that the ISC was complex and needed proactive leadership, united command, prioritizing the COVID-19 pandemic, powerful supervising, coordination between health and medical sections, and strong social capital. Participants stated that *leadership in management of COVID-19 pandemic* was extremely important and challenging, especially in the early stages of the pandemic, that ISC should be implemented. Participants also believed that *united command* was important to fruitfully implement the ISC: *“Trusteeship is a big challenge. One of the problems in our country is that the Ministry of Health is considered the custodian of health, while the custodian of health in all communities must be with the highest executive authority in the country. There is no united command with sufficient authority in this field (COVID-19 pandemic).” (Participant at MOHME with 28 years of work experience; Male; 54 years old).*

Some participants stated that the COVID-19 pandemic was not *prioritized* over other social, economic, and political issues at the government level, that made it an important challenge for ISC to improve the response to the pandemic: *“That we cannot have cross-sectoral coordination is important; however, the more important issue is that the related governing body still does not consider the issue of COVID-19 as main health priority.” (Participant at University with 20 years of work experience; Male; 50 years old).*

The participants reported lack of adequate *intra-sector collaboration* as another challenge: *“Unfortunately, we do not have enough intra-sector collaboration. This is very important. When an organization is not coordinated within itself, it will definitely not collaborate successfully with other organizations. This was also seen in the COVID-19 pandemic.” (Participant at University with 25 years of work experience; Female; 48 years old).*

Also, poor *supervising* on performance of organizations slowed down ISC: *“Supervising is not done well; sometimes contradictory messages about Corona are broadcasted to people by various organizations, for example, Organization X provides information that is not approved by Organization Y.” (Participant at MOHME with 27 years of work experience; Male; 56 years old).*

Poor *collaboration between health and medical sections* was also a big challenge stated by the participants that inhibited improving the response to the COVID-19 pandemic from the participants’ points of views: *“I think the main health issue in our country is the separation between health and medical sections. This issue was also evident in the COVID-19 pandemic and we were hit by it.” (Participant at Hospital with 4 years of work experience; Male; 42 years old).*

The participants also stated that poor *social capital* could be a challenge in implementation of ISC: *“We must strengthen the social capital. Individuals’ distrust, organizations’ distrust, as well as the government’s distrust is important barriers to participation and collaboration.” (Participant at University with 22 years of work experience; Male; 64 years old).*

### Domain 2: outer settings

In this domain, the participants identified networking with external organizations/ cosmopolitanism as a key challenge for successful implementation of inter-sectoral efforts to improve the response to COVID-19 pandemic.

#### Networking with external organizations

Cosmopolitanism refers to the degree to which an organization is networked with other external organizations [22]. The participants referred to building relationships with local organizations and using their capacities, however they did not mention international collaboration. Communication with local organizations has been more about exchanging information, providing advice, developing guidelines, and providing personal protective equipment such as masks: *“We also liaised with other organizations such as national television, newspapers, municipalities, and education sectors. We gave them guidelines, and tried to use the capacities that existed in these organizations to inform the people.” (Participant at University with 19 years of work experience; Male; 52 years old).*

### Domain 3: inner setting

In inner setting domain, the participants identified culture, available resources, and access to knowledge and information as key challenges for the successful implementation of inter-sectoral efforts to improve the response to COVID-19 pandemic.

#### Culture

The culture in this domain reflects the norms, values, and basic assumptions of an organization regarding the intended intervention [22]. The participants referred to the beliefs of managers and officials of organizations about ISC as well as non-institutionalization of the culture of ISC in organizations: *“Manager and officials’ beliefs in collaboration are important prerequisites for its successful implementation .... Some managers do not believe in collaboration, they find it disturbing the routine actions of their organization.” (Participant at MOHME with 10 years of work experience; Female; 40 years old).*

#### Available resources

Available resources refer to the level of resources dedicated for implementation and on-going operations, including money, training, education, physical space, and time [22]. Resources are not unlimited; however using them properly can increase the effectiveness of any intervention. The participants in this study stated that financial resources were not major concern in this regard and had the least impact on limitation of ISC. The important point was the management of these resources: “*… If the two organizations do not work together, it is not because of limited resources. There is always resources limitation, it is important to prioritize and redistribute resources.” (Participant at University with 25 years of work experience; Female; 48 years old).*

#### Access to knowledge and information

Ease of access to understandable information and knowledge about the intervention and how to combine it in work tasks were other important constructs related to the inner setting of the intervention that were mentioned by the participants. The participants believed that to create the ISC, sufficient knowledge about the collaboration was needed to enable them to have interactions with other different organizations to control the Covid-19: *“We need to know how to interact with other organizations to manage a crisis like the COVID-19 pandemic.” (Participant at Hospital with 12 years of work experience; Male; 42 years old).*

### Domain 4: characteristics of the individuals

This domain included knowledge and beliefs related to the intervention.

#### Knowledge and beliefs about the intervention

Individuals’ attitudes toward and value placed on the ISC as well as familiarity with the facts, truths, and principles related to the collaboration intervention were important characteristics of the individuals who were involved in the implementation of ISC in improving response to the COVID-19 pandemic.

*“One of the basic foundations of crisis management is inter-sectoral collaboration. Without it, there is no possibility of success.” (Participant at MOHME with 28 years of work experience; Male; 54 years old).*

### Domain 5: process of implementation

In this domain, the participants stated that planning ISC implementation as an important measure for successful implementation of the intervention.

#### Planning

The lack of a clear and comprehensive structure and program of ISC within the organizations themselves and the government level, especially in the early outbreak of the pandemic was raised as a challenge in this regard: *“ISC is not spontaneously generated. It needs a proper structure and program. We had poor collaboration planning for the management of COVID-19, especially at the beginning of the pandemic.” (Participant at University with 32 years of work experience; Male; 56 years old).*

In addition, as noted before, lack of a strong command that could assist ISC between different organizations to improve pandemic response equally hindered the design of such an intervention. Formation of the National Corona Headquarter and its provincial branches were examples of collaboration between different departments in order to respond to the pandemic, however there were implementation challenges in this regard.

## Discussion

Inter-sectoral/ organizational collaboration is key approach in controlling epidemics. This study was conducted with the aim of exploring the challenges for implementation of inter-sectoral efforts to improve COVID-19 pandemic response in Iran using CFIR. Findings showed the challenges from different levels viewpoints of experts (MOHME, INIHR, medical universities and hospitals). In summary, the findings show that the ISC approach in all five CFIR domains (ISC characteristics, outer setting, inner setting, characteristics of individuals and process of implementation) needs to be improved. These findings can be used as lessons learned for managing future health crises.

Regarding *intervention source*, participants reported that assigning responsibilities from upstream organizations to make ISC was rarely clear to most participants working in the field. This challenge was highlighted in other studies too. For example, Shushtari in their study in 2022 showed lack of coherent policy and poor collaboration between the health sector and other sectors involved in managing the pandemic [27].

At the public level, *leadership and united command system, priority of the COVID-19 pandemic, intra-sector collaboration, supervisory of the organizations activities, and social capital* were important challenging factors that showed complexity of ISC implementation. In crises, in order to create coordination and prevent the interference of tasks and functions, it is necessary for all operational units to provide services under a *single command* [28]. In the COVID-19 pandemic in Iran, the main responsibility for controlling the pandemic was vested in the MOHME, while in practice it lacked sufficient executive power. Over time (about eight months after the onset of the pandemic) and due to the pervasive nature of the problem, the command system of the country was formed under the name of “National Corona Headquarter”, which created a more cohesive management. The presence of committed experts with sufficient specialized knowledge in the crisis management was vital in this headquarters to help them make right decisions. Similar findings have been reported, according to which a lack of application of the whole-government approach, not only ministries of health, especially at the beginning of the COVID-19 pandemic has been an important challenge [29]. Similarly, in another study conducted in Nigeria, lack of transparency, *lack of leadership*, and civil disobedience were identified as major factors of failure in the fight against COVID-19 [30]. However, success in managing the COVID-19 pandemic following strong, committed, and accountable central leadership has also been shown in other studies in Vietnam, New Zealand, and South Korea [31], Taiwan [32], and the United Arab Emirates [33].

Another challenge stated by the participants was that the COVID-19 pandemic seemed not to be a *priority* for the government and policymakers. The results of Shushtari et al.’s study also showed that one of the major challenges in managing the COVID-19 crisis was the government’s lack of serious attention to the disease and the delay in decision-making [27]. In a study by Omidi et al., it was also mentioned that the unknown nature of the virus and lack of beliefs in its vulnerability played an important role in rapid spread of the disease in the country [34]. In this study, *intra-sector collaboration* was mentioned as another challenge by the participants. Weakness in intra- and inter-organizational collaboration as the main challenge of health sector in disaster management has been confirmed in other studies [35–38]. In the study of Bijani et al., intra- and inter-organizational coordination was reported as the main weakness of COVID-19 pandemic management [35]. Also, another study that analyzed the ISC in the Iranian health system for implementing health in all policies showed that the MOHME did not pursue coordination policies between its subordinate deputies, and this issue was not far from the eyes of other agencies and people’s representatives, and was a serious obstacle to collaboration [36].

*Supervising the related organizations’* activities was another challenge for ISC from the participants’ point of view. In a study by Mirkazehi Rigi et al., they examined the challenges and strategies to deal with COVID-19 from the perspective of physicians and nurses. It was recommended to develop a comprehensive procedure for monitoring the performance of COVID-19 pandemic crisis management in Iran [39]. However, a strong monitoring system in Hong Kong, Singapore and Japan helped these countries have a good response to the COVID-19 control [40]. Furthermore, *social capital* was mentioned as an important and challenging issue for ISC by the participants. Social capital is a significant asset for individuals, communities and their governments, especially in times of crisis [41]. Undoubtedly, controlling the COVID-19 pandemic requires people’s trust in government and their participation [42]. Wong and Jensen (2020) in their study showed that public trust in political leaders in the face of risks was recognized as one of the important components of effective and efficient risk management. However, in the face of a pandemic like COVID-19, trust could turn into a double-edged sword. This means that in such a condition, public trust in the competence and care of the government may lead people to underestimate the risks of disease. On the other hand, people’s distrust of the government may reduce their care measures [43]. A study by Hsia et al. (2020) found that factors such as transparency in the policy-making process, information updates, and access to social infrastructure helped increasing public trust in the government in COVID-19 pandemic [44]. Chatzopoulou study also showed that the existence of social capital could facilitate the implementation of government programs, especially the closure of various centers and social distancing [45].

According to our participants, the *main relationship of organizations* was about exchanging information, developing protocols and guidelines, consulting and providing medical services. In the study by Turner et al., the collaboration of organizations adjacent to the health system (universities and private sectors) to COVID-19 pandemic response, included providing evidence for decision-making, service capacity and supporting coordination [16].

The *beliefs of managers and officials, non-institutionalization of the culture of ISC*, and *access to knowledge and information towards implementation of inter-sectoral efforts* were reported as important challenges in domains of individual characteristics and inner setting. The study participants’ report showed that the beliefs of managers and officials of related organizations about ISC were limited. Belief in ISC and commitment to health-centeredness for those involved does not occur spontaneously, and action in this area is necessary. Establishing an accreditation and rating system can be a practical strategy to this behavior change [36]. *Lack of sufficient knowledge and information about ISC* amongst the management body was another challenge identified in our study. In the study of Damari et al., lack of knowledge, attitude and skills towards ISC among experts and managers of MOHME and universities was expressed as a challenge to implement it [36]. The results of Bijani et al.’s study also showed that familiarity with the principles of teamwork is essential for managing the COVID-19 pandemic [35].

*The lack of specific structure and programs for collaboration* within the organizations themselves and at government level, especially in the early stages of the pandemic, was identified as a challenge related to the planning construct. As reported in previous research, lack of proper and coherent planning, lack of crisis management, and lack of coordination between organizations, policy makers, experts and officials were important barriers to control COVID-19 pandemic [39–41, 43, 44, 46]. Demari et al. also showed in their study that lack of a clear vision and a comprehensive plan for ISC in the health system has caused the expectations of the health sector from other departments to be unclear [36]. The study participants believed that paying attention to the capacities and structures in the health system and applying the experiences and lessons learned in crisis management is vital in improving pandemic response. According to the participants, capacity and structure of related offices in MOHME were not used properly in the COVID-19 pandemic. It shows necessity of strengthening their structures to predict, prepare for, and combat communicable diseases. Bagheri Lankarani et al. reported in their study that important opportunities to control COVID-19 pandemic were lost due to neglect of the country’s capacities and lack of proper foresigh [47]. Some participants also noted that the issue of cost in ISC was not a major problem compared to the challenges of planning, monitoring and coordination.

In this study, we used COVID-19 experience to identify challenges for implementation of inter-sectoral efforts to improve outbreak response. However, there might be other outbreaks as well as global crises like climate disasters that need ISC to be controlled and managed. The challenges identified in our study that was categorized using CFIR can be used to shed light on other society’s responses to other crisis. Through this study, we learned that we have challenges in intervention characteristics, outer setting, inner setting, characteristics of individuals, and process of implementation of ISC efforts to improve COVID-19 response. The lessons/insights of this study could be relevant to current and future research in years to come, conducted in Iran or other similar contexts.

### Study strengths and limitations

As far as we know, this is the first study to examine the challenges for implementation of inter-sectoral/ trans-disciplinary efforts to improve COVID-19 pandemic response in Iran from the perspective of experts at various levels of the health system. Our qualitative study was conducted using CFIR - one of the most important frameworks in the field of IS - which helps to understand, describe and identify factors that contribute to the further success of implementation. Also, recruitment of participants from different levels of health care system helped to investigate the issue from different levels. However, in order to better understand the issue, these challenges should be investigated from the perspectives of experts in other sectors and organizations.

## Conclusions

In summary, our study showed that leadership and command system, prioritizing the COVID-19 problem, intra-sector collaboration, adequate supervisory, coordination between health and medical sectors, social capital, managers and officials beliefs, culture of ISC, sufficient knowledge and information about ISC, and specific structures and programs for ISC were important and challenging issues to implement ISC approach to improve COVID-19 pandemic response. These findings recall the need to develop and in particular, implement a specific structure and program at the government level to strengthen this approach.

Improving ISC need to increase managerial knowledge. Also, it is necessary to take measures to improve organizational culture in order to increase ISC. It is also recommended that lessons learned from the successes and failures of ISC be recorded and stored for use in future probable epidemics and potential crises. Changing the attitude of officials towards the impact and synergy of ISC is also an important issue to be considered.

## Supplementary Information

**Additional file 1.**

**Additional file 2.**

## Data Availability

The datasets generated and analyzed during the current study are not publicly available due to privacy restrictions of the participants but are available from the corresponding author on reasonable request.

## Abbreviations

EIDs: Emerging Infectious Diseases
COVID-19: Coronavirus Disease 2019
MOHME: Ministry of Health and Medical Education
SARS: Severe Acute Respiratory Syndrome
ISC: Inter-sectoral Collaboration
WHO: World Health Organization
EU: European Union
IS: Implementation Science
CFIR: Consolidated Framework for Implementation Research
COREQ: Consolidated Criteria for Reporting Qualitative Research
INIHR: Iranian National Institute of Health Research

## References

[1] Tabish SA (2020). COVID 19: the worst health calamity of the world. Int J Sci Res.

[2] Zu ZY, Jiang MD, Xu PP, Chen W, Ni QQ, Lu GM, Zhang LJ (2020). Coronavirus disease 2019 (COVID-19): a perspective from China. Radiology.

[3] World Health Organization: coronavirus disease 2019 (COVID-19): situation report.Geneva, 2022. Available from: https://covid2019.who.int/. 35767666

[4] Islamic Republic of Iran Ministry of Health and Medical Education (MOHME); 2020. Available from: http://ird.behdasht.gov.ir.

[5] Schneider H, Okeyo IL, Du Toit A, Engelbrecht B, London L, Pegram E, Reagon G, Cloete K (2021). Intersectoral collaboration before and during the COVID-19 pandemic in the Western cape: implications for future whole-of-society approaches to health and wellbeing. South Afr Health Rev.

[6] Damari B, Vosoogh Moghaddam A (2014). Improving approaches of intersectoral collaboration for health by health and food security high council in IR Iran. J School Public Health Institute Public Health Res.

[7] Adeleye OA, Ofili AN (2010). Strengthening intersectoral collaboration for primary health care in developing countries: can the health sector play broader roles?. J Environ Public Health.

[8] World Health Organization: Intersectoral action for health: a cornerstone for health-for-all in the twenty-first century.:Report to the International Conference; Halifax, Nova Scotia, Canada; 20–23 April 1997.

[9] Stahl T, Wismar M, Ollila E, Lahtinen E, Leppo K: Health in all policies: prospects and potentials. Helsinki: Finnish Ministry of Social Affairs and Health.2006. Available from: https://www.euro.who.int/__data/assets/pdf_file/0003/109146/E189260.pdf.

[10] Maher A, Malmir R, Toghyani R, Safari MS (2020). COVID-19 crisis management: reengineering the health care system in Iran. Journal of medical Council of Iran.

[11] Samaan G, McPherson M, Eidman J, Obubah O, Baptiste J-P, Kuppens L, et al. The World Health Organization's actions within the united nations system to facilitate a whole-of-society response to COVID-19 at country level. Front Public Health. 2021;9. 10.3389/fpubh.2021.831220. 10.3389/fpubh.2021.831220 PMC8804283 35118047

[12] World Health Organization: COVID-19 Strategy.:https://www.who.int/emergencies/diseases/novel-coronavirus-2019/strategies-and-plans. Updated April 2020.

[13] Legido-Quigley H, Asgari N, Teo YY, Leung GM, Oshitani H, Fukuda K, Cook AR, Hsu LY, Shibuya K, Heymann D (2020). Are high-performing health systems resilient against the COVID-19 epidemic?. Lancet.

[14] Tell D, Oldeide O, Larsen T, Haug E (2022). Lessons learned from an intersectoral collaboration between the public sector, NGOs, and sports clubs to meet the needs of vulnerable youths. Societies.

[15] Li B, Huikuri S, Zhang Y, Chen W (2015). Motivating intersectoral collaboration with the hygienic city campaign in Jingchang, China. Environ Urbanization.

[16] Turner S, María Ulloa A, Niño N, Valencia Godoy V. The role of intersectoral action in response to COVID-19: a qualitative study of the roles of academia and the private sector in Colombia. Int J Health Policy Manag. 2021:1–13 10.34172/IJHPM.2021.100. 10.34172/ijhpm.2021.100 PMC9808252 34523858

[17] Mlozi MR, Rumisha SF, Mlacha T, Bwana VM, Shayo EH, Mayala BK, et al. Challenges and opportunities for implementing an intersectoral approach in malaria control in Tanzania. Tanzania J Health Res. 2015;17(1). 10.4314/thrb.v17i1.2.

[18] Wang H, Qi H, Ran B (2022). Public–private collaboration led by private organizations in combating crises: evidence from China’s fighting against COVID-19. Admin Soc.

[19] Rantala R, Bortz M, Armada F (2014). Intersectoral action: local governments promoting health. Health Promot Int.

[20] Livet M, Haines ST, Curran GM, Seaton TL, Ward CS, Sorensen TD, Roth McClurg M (2018). Implementation science to advance care delivery: a primer for pharmacists and other health professionals. Pharmacotherapy: the journal of human pharmacology and drug. Therapy.

[21] Eccles M, Mittman B (2006). Welcome to implementation science. Implement Sci.

[22] Damschroder LJ, Aron DC, Keith RE, Kirsh SR, Alexander JA, Lowery JC (2009). Fostering implementation of health services research findings into practice: a consolidated framework for advancing implementation science. Implement Sci.

[23] Hutchinson SL, Lauckner H, Gallant KA, Stilwell C, Meisner BA (2019). What does it take to build sustainable intersectoral recreation initiatives? Learning from the consolidated framework for implementation research (CFIR). Leisure/Loisir.

[24] Tong A, Sainsbury P, Craig J (2007). Consolidated criteria for reporting qualitative research (COREQ): a 32-item checklist for interviews and focus groups. Int J Qual Health Care.

[25] Consolidated Framework for Implementation Research: .Available from: http://www.cfirguide.org/. Assessed on 12 Aug 2020.

[26] Lincoln YS, Guba EG (1985). Naturalistic inquiry.

[27] Jorjoran Shushtari Z, Shirazikhah M, Ahmadi S, Salimi Y, Biglarian A, Almasi A, Paykani T (2022). Upstream determinants and downstream risk factors of COVID-19 infection in Iran: a qualitative study of health professionals’ views. J Health Sci Surveillance Syst.

[28] Mehrabi Tavana A (2020). Lessons learned from the coronavirus disease pandemic. J Culture Health Promotion.

[29] Raoofi A, Takian A, Sari AA, Olyaeemanesh A, Haghighi H, Aarabi M (2020). COVID-19 pandemic and comparative health policy learning in Iran. Arch Iranian Med (AIM).

[30] Uroko FC, Nwaoga CT (2021). Fighting COVID-19 in Nigeria: leadership and collaboration in numbers 12: 9-16. Verbum et Ecclesia.

[31] Bhalla A. Leadership challenges and the COVID-19 pandemic. ORF. Occas Pap. 2021;299.

[32] Hsieh C-W, Wang M, Wong NW (2021). Ho LK-k: a whole-of-nation approach to COVID-19: Taiwan’s National Epidemic Prevention Team. Int Polit Sci Rev.

[33] Zaher WA, Ahamed F, Ganesan S, Warren K, Koshy A. COVID-19 crisis management: lessons from the United Arab Emirates leaders. Frontiers. Public Health. 2021;9. 10.3389/fpubh.2021.724494. 10.3389/fpubh.2021.724494 PMC8585940 34778167

[34] Omidi M, Maher A, Etesaminia S (2020). Lessons to be learned from the prevalence of Covid-19 in Iran. Med J Islamic Republic of Iran.

[35] Bijani M, Karimi S, Khaleghi A, Gholampoor Y, Fereidouni Z (2021). Exploring senior managers’ perceptions of the COVID-19 crisis in Iran: a qualitative content analysis study. BMC Health Serv Res.

[36] Damari B, Vosough MA, Rostami GN, Salarianzadeh M, Malek Afzali S (2020). Analysis of intersectoral collaboration in the Iranian health system for implementing health in all policies: challenges and the way forward. J School Public Health Institute Public Health Res.

[37] Bahadori M, Khankeh HR, Zaboli R, Ravangard R, Malmir I: Barriers to and facilitators of inter-organizational coordination in response to disasters: a grounded theory approach. Dis Med Public Health Preparedness 2017, 11(3):318–325. http://dx.doi.org/10.1017/dmp.2016.131. 10.1017/dmp.2016.131 27725007

[38] Bahadori M, Khankeh HR, Zaboli R, Malmir I (2015). Coordination in disaster: a narrative review. Int J Med Rev.

[39] Mirkazehi Rigi Z, Dadpisheh S, Sheikhi F, Balouch V, Kalkali S (2020). Challenges and strategies to deal with COVID-19 from the perspective of physicians and nurses in southern of Sistan and Baluchestan, Iran. J Military Med.

[40] Pooladi M, Entezari M, Hashemi M, Bahonar A, Hushmandi K, Raei M (2020). Investigating the efficient management of different countries in the COVID-19 pandemic. J Marine Med.

[41] Damghanian H, Keshavarz M (2020). The effect of Covid-19 disease on political trust with regard to the mediating role of social capital and the moderating effect of social media. J Government Stud.

[42] Labaf A, Jalili M, Jaafari Pooyan E, Mazinani M (2021). Management of Covid-19 crisis in Tehran University of Medical Sciences hospitals: challenges and strategies. J School of Public Health Institute Public Health Res.

[43] Wong CML, Jensen O (2020). The paradox of trust: perceived risk and public compliance during the COVID-19 pandemic in Singapore. J Risk Res.

[44] Hsia C (2020). Government intervention or social innovation: understand how Taiwan is coping with COVID-19 and developing resilience through its social infrastructure services. J Global Res.

[45] Chatzopoulou S. Social trust and government government responses to COVID-19. Soc Eur. 2020; Available from: https://socialeurope.eu/social-trust-and-government-responses-to-covid-2019.

[46] Tajvar A, Hosseini Z, Farahbakhsh M, Fakherpour A (2021). Explaining the challenges of coping with coronavirus crisis in the workplaces: a qualitative study.

[47] Bagheri Lankarani K, Khayamzadeh M (2021). Points for improving the COVID-19 national control program. J Culture Health Promot.

