# Supplementary material for: Challenges for implementation of inter-sectoral efforts to improve outbreak response using consolidated framework for implementation research; Iran’s COVID-19 experience
Source: BMC Health Serv Res. 2022 Sep 3;22:1118. doi: 10.1186/s12913-022-08510-4 (PMC9440312; doi:10.1186/s12913-022-08510-4)
Supplement: Supplementary file 2 — Additional file 2. [file 12913_2022_8510_MOESM2_ESM.docx]

**ANNEX 2- Interview Guide**

**Research question:** What are the challenges of implementing inter-sectoral/ organization efforts to improve COVID-19 pandemic response in Iran?

**Interview questions:**

- How did your organization get involved in the COVID-19 pandemic?
- Were your organization's efforts to build inter-sectoral/ organizational collaborate to respond to the pandemic announced from the outside or was it a spontaneous and voluntary action?
- In your opinion, how much implementing inter-sectoral/ organizational collaboration is possible in our country to improve the response to the COVID-19 pandemic?
- In your opinion, how difficult is it to implement inter-sectoral / organization efforts to improve COVID-19 pandemic response? What are the challenges?
- Have you collaborated with other organizations to improve COVID-19 pandemic response?
- If you have collaboration with other organizations, what was the quality of this relationship? How successful do you think it was?
- In your opinion, are the norms, values and practices of your organization consistent with inter-sectoral/ organizational collaboration?
- Do you have adequate resources (money, personnel, physical space and time) in your organization to implementation inter-sectoral/ organizational collaboration?
- In your opinion, how necessary and effective is the implementation of inter-sectoral/ organizational collaboration to improve COVID-19 pandemic response?
